# Efficacy of Small Incision Cataract Surgery: A Multicenter Retrospective Study of Visual Outcomes in Coastal Ecuador

**DOI:** 10.3390/vision9030060

**Published:** 2025-07-15

**Authors:** Roberto Ernesto Alcívar-Viteri, Verónica Dolores Moreira-Pico, Carlos Iván Gómez-Cedeño, Julia Patricia Duran-Ospina, Aline Siteneski, Karime Montes-Escobar

**Affiliations:** 1 Instituto de la Visión, Portoviejo 130105, Ecuador; veronicamoreirapico@hotmail.com; 2 Departamento de Matemáticas y Estadística, Facultad de Ciencias Básicas, Universidad Técnica de Manabí, Portoviejo 130105, Ecuador; cgomez3097@utm.edu.ec; 3 Faculty of Health Sciences, Optometry Career, Universidad Técnica de Manabí, Portoviejo 130105, Ecuador; julia.duran@utm.edu.ec; 4 Dirección de Investigación, Carrera de Medicina, Facultad de Ciencias de la Salud, Universidad Técnica de Manabí, Portoviejo 130105, Ecuador; aline.siteneski@gmail.com; 5 Department of Statistics, University of Salamanca, 37008 Salamanca, Spain

**Keywords:** Small Incision Cataract Surgery (SICS), visual acuity, low- and middle-income countries, cataract surgery outcomes, ocular comorbidities, avoidable blindness

## Abstract

Cataracts remain one of the leading causes of reversible blindness in low- and middle-income countries such as Ecuador. This study assessed the efficacy of Small Incision Cataract Surgery (SICS) and analyzed sociodemographic and clinical factors associated with postoperative visual outcomes. A retrospective multicenter analysis was conducted across six ophthalmology clinics along the Ecuadorian coast between 2023 and 2024, including 558 patients aged 30 years or older. Postoperative visual acuity, measured using the LogMAR scale, improved significantly (mean improvement of 0.525 LogMAR units in the right eye (OD) and 0.489 LogMAR units in the left eye; *p* < 0.001). Ages between 60 and 69 years were associated with better outcomes in the right eye, while male sex was a protective factor against poor visual acuity in the left eye. Although diabetes mellitus and hypertension were prevalent, neither condition showed a significant association with postoperative visual outcomes. The findings confirm that SICS is a safe, effective, and cost-efficient surgical approach for restoring vision in resource-limited settings, supporting its inclusion in national public health strategies to reduce avoidable blindness in developing countries.

## 1. Introduction

Cataract is one of the leading causes of reversible blindness and visual impairment. Older adults are the most vulnerable population affected by cataracts [1,2]. According to the World Health Organization [3], approximately 94 million people worldwide are affected by cataracts, representing over 50% of all cases of preventable blindness [4]. In low- and middle-income countries, the limited access to ophthalmological services, insufficient surgical capacity, and long waiting lists intensify the disease burden [5] Globally, advances in surgical technology, especially the widespread adoption of phacoemulsification, have significantly improved visual recovery outcomes [6]. Economic constraints, geographic barriers, and a shortage of trained professionals hinder universal surgical coverage, particularly in rural and underserved areas [7,8,9,10].

The prevalence of cataracts has increased over time in Latin America [6,11,12]. Paraguay revealed that untreated cataracts are responsible for between 41% and 87% of blindness cases [13]. Despite the progress of cataract in Latin American countries, they still face limited surgical access and inconsistent postoperative outcomes [14]. The suboptimal surgical results of cataract may be linked to socioeconomic conditions and systemic constraints [15]. Similarly, a national survey in Argentina indicated that, despite public policy advances, avoidable blindness remains a pressing issue [16]. These findings underscore the need for reliable regional data to inform public health strategies for visual care [17].

The SICS technique is a low-cost, sutureless procedure that is frequently used safely and efficiently, particularly in older adults [18]. It offers visual outcomes comparable to phacoemulsification, enabling large-scale sight restoration without advanced surgical equipment [19]. Beyond its clinical efficacy, SICS contributes to social reintegration by enhancing functional independence and quality of life in individuals affected by preventable blindness. Recently, global initiatives such as *Vision 2020: The Right to Sight*, led by the WHO, prioritize interventions to reduce the global burden of avoidable blindness [20].

In Ecuador, cataracts represent a significant public health challenge due to the limited access to ophthalmological services, a shortage of trained professionals, and significant geographic barriers [21,22]. The country faces specific challenges such as an aging population and prolonged exposure to ultraviolet radiation, particularly in rural and coastal zones, which exacerbate cataract progression [23]. As a result, a substantial number of individuals suffer from preventable blindness, with a high prevalence in older adults, who are the most vulnerable demographic for cataract development [1].

The SICS technique has emerged as an effective and cost-efficient surgical option, particularly in resource-limited settings. Unlike phacoemulsification, which requires advanced surgical equipment, SICS is a low-cost, sutureless technique that can be performed with minimal equipment, making it ideal for high-volume surgical programs [18,24]. Several studies conducted in middle-income countries, including Nepal and India, have demonstrated the success of SICS in improving visual outcomes and restoring functional vision in cataract patients [24,25]. Despite the demonstrated efficacy of this technique, there remains a gap in understanding its specific impact in Ecuadorian settings, where access to eye care services is often limited.

In Ecuador, the National Institute of Statistics and Census reports that cataracts are one of the primary causes of hospital-based ophthalmologic morbidity [22]. The Ministry of Public Health further highlights the correlation between cataracts and systemic conditions such as diabetes mellitus and hypertension, which are prevalent in the Ecuadorian population and contribute to the progression of cataract formation [23]. Given these conditions, the need for a comprehensive study of SICS in the Ecuadorian context is critical to inform public health strategies and optimize the use of available resources to combat preventable blindness.

**Figure 1:**
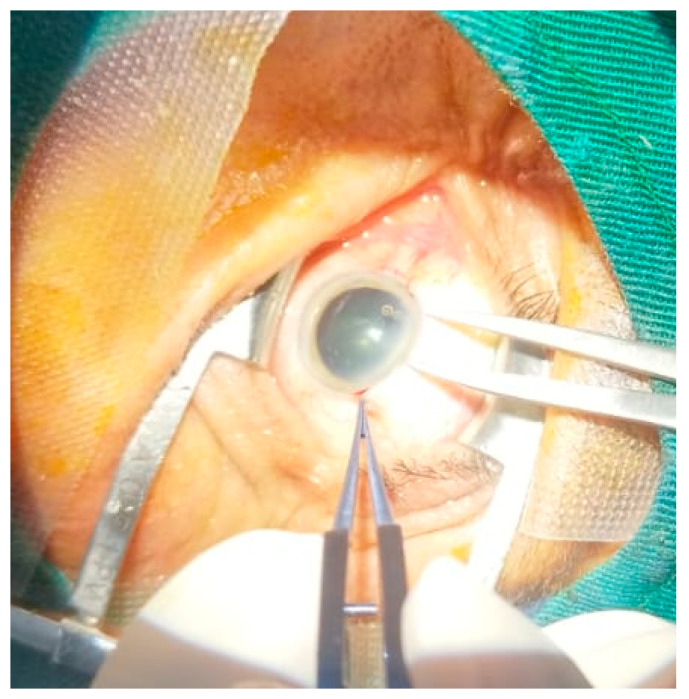
Small Incision Cataract Surgery (SICS) technique at the Institute of Vision: ongoing surgical procedure.

This study is particularly needed as there is limited research on the specific impact of SICS in the Ecuadorian context, where access to ophthalmological care is often restricted. While SICS has been widely studied in other low- and middle-income countries, there remains a gap in understanding its effectiveness in Ecuador, where unique challenges such as an aging population, chronic UV exposure, and systemic comorbidities like diabetes and hypertension are prevalent. This research aims to fill this gap by assessing the outcomes of SICS in improving postoperative visual acuity among patients from coastal Ecuador. Additionally, it will provide valuable insights that can help optimize cataract surgery practices in Ecuador, inform public health strategies, and contribute to reducing preventable blindness in underserved regions. By focusing on this population, the study highlights the potential of SICS as a sustainable and cost-effective intervention tailored to Ecuador’s specific healthcare needs. The novelty of this study is emphasized as the first multicenter analysis of cataract surgery in the Ecuadorian coastal region. This work provides valuable regional evidence to inform visual health policies in low-resource settings (Figure 1).

## 2. Materials and Methods

### 2.1. Study Design

A descriptive, retrospective, multicenter study with a multivariate approach was conducted between 1 January 2023 and 31 December 2024 within the institutional framework of Instituto de la Visión S.A. (Ophthalmology and Eye Surgery Center). The six ophthalmology clinics are located in Manta, Santo Domingo, Quevedo, Guayaquil, Daule, and Portoviejo. The latter served as the institution’s main headquarters and was responsible for centralizing data collection and systematization. The study sample consisted of 558 patients who underwent cataract surgery at one affiliated clinic during the specified period.

The study included patients who underwent cataract surgery in both eyes at the clinics of Instituto de la Visión S.A. between January 2023 and December 2024. The inclusion criteria were as follows: (1) patients with complete clinical records containing data on age, sex, residence area, comorbidities, surgical technique used, and preoperative and postoperative visual acuity; (2) only cataract surgeries, without the combination of other ocular procedures; (3) bilateral cataract surgeries; and (4) individuals aged 30 and older, up to 90 years. We excluded patients who underwent surgery in only one eye, those under 30 years of age, and those with incomplete or inconsistent clinical records, particularly those lacking postoperative data or information on comorbidities, or where the surgical technique used was not identified.

All patients were treated exclusively using the SICS technique. This procedure is widely recognized for its safety, low cost, and effectiveness, especially in resource-limited settings. The technique involves a self-sealing sclerocorneal tunnel incision of approximately 6–7 mm, followed by manual expression of the lens nucleus and implantation of a posterior chamber intraocular lens (PCIOL). It does not require a phacoemulsification machine and can be performed with minimal equipment, making it ideal for high-volume surgical programs. While the majority of surgeries were uneventful, some minor postoperative complications were observed, including mild inflammation and transient elevated intraocular pressure. These complications were promptly managed according to clinical protocols. No major complications, such as endophthalmitis or significant vision loss, were reported.

All surgeries were performed by experienced ophthalmic surgeons under local anesthesia, following strict aseptic protocols. Visual acuity was assessed using the LogMAR scale, in accordance with the guidelines established by the World Health Organization (WHO) and the RAAB (Rapid Assessment of Avoidable Blindness) methodology. For analytical purposes, three clinical categories were defined: “Good” vision (LogMAR ≤ 0.3, equivalent to 6/12 or better); “Moderate” or “Regular” vision (LogMAR > 0.3 and ≤1.0, equivalent to <6/12 to 6/60); and “Poor” vision (LogMAR > 1.0), considered functional blindness.

### 2.2. Statistical Analysis

This classification facilitated an objective evaluation of the level of visual disability and the analysis of associated clinical and demographic variables. Data processing and analysis were carried out using the Statistical Package (SPSS) version 24. Prior to the application of parametric tests, the assumptions of normality were assessed using the Shapiro–Wilk test, confirming the appropriateness of using parametric approaches for the comparison of visual acuity values.

Descriptive statistics were used to summarize frequencies and percentages. Bivariate analysis was performed using the Chi-square test. Paired-sample *t*-tests were applied to compare pre- and postoperative LogMAR values. One-way ANOVA was used to assess differences across age groups. In addition, multivariate analysis was conducted through multinomial logistic regression to identify clinical and demographic predictors associated with postoperative visual outcomes (good, regular, or poor), using visual acuity (LogMAR category) as the dependent variable.

Additionally, a scatter plot analysis was conducted to visually evaluate the relationship between preoperative and postoperative visual acuity in both eyes. This graphical method allowed the identification of trends and outliers by plotting individual-level LogMAR values before and after surgery.

## 3. Results

Of the 558 patients who underwent cataract surgery and were evaluated, the gender distribution was nearly balanced, with 50.2% males (*n* = 280) and 49.8% females (*n* = 278). No statistically significant differences were found in prevalence between sexes for the (OD) (χ^2^ = 0.375; df = 2; *p* = 0.829), indicating a homogeneous distribution by gender. The average age of patients was concentrated in older age groups, with the majority between 70 and 79 years (46.6%, *n* = 260), followed by the 60 to 69 years group (28.9%, 61), while a small percentage corresponded to patients younger than 60 years. A statistically significant association was observed between age group and postoperative visual acuity in the (OD) (χ^2^ = 20.03; df = 10; *p* = 0.029), suggesting that age is a relevant factor influencing surgical outcomes. However, this association did not reach statistical significance in the left eye (*p* = 0.058), although it showed a trend toward significance.

Regarding geographic origin, most patients came from urban areas (62.0%, *n* = 346) compared to rural areas (38.0%, *n* = 212). Nonetheless, the place of residence showed no statistically significant relationship with postoperative visual outcomes in either eye (*p* > 0.05). Additionally, a high prevalence of arterial hypertension (42.5%, *n* = 237) and diabetes mellitus (66.3%, *n* = 370) was recorded in the study population. However, neither comorbidity showed a statistically significant correlation with postoperative visual acuity in the (OD) (χ^2^ = 4.49; df = 2; *p* = 0.106 for diabetes), suggesting that, under the conditions analyzed, these pathologies did not significantly affect postoperative visual outcomes (Table 1).

A paired-samples *t*-test was conducted to compare preoperative and postoperative visual acuity in both eyes, revealing highly significant improvements that indicate a substantial clinical benefit from cataract surgery. This statistical test accounts for the repeated measurements within the same individuals, strengthening the validity of the findings by controlling for intra-subject variability.

### More Results

In the (OD), the mean difference in LogMAR visual acuity before and after surgery was 0.525 units (*t* = 24.872; degrees of freedom = 557; *p* < 0.001). The 95% confidence interval ranged from 0.484 to 0.567, indicating that the observed improvement was both consistent and precise across the patient cohort. This magnitude of change corresponds to an improvement of approximately five lines on a standard visual acuity chart, which is clinically meaningful and translates into a substantial gain in patients’ functional vision.

Similarly, in the left eye (OI), the mean LogMAR improvement was 0.489 units (t = 23.251; df = 557; *p* < 0.001), with a 95% confidence interval between 0.448 and 0.531. These results further confirm that the surgical intervention produces a reliable and significant enhancement in visual performance bilaterally.

Overall, these findings demonstrate that cataract surgery leads to a statistically robust and clinically important improvement in visual acuity, with an average gain close to half a LogMAR unit in both eyes. The *p*-values below 0.001 allow for a confident rejection of the null hypothesis, affirming that the improvements observed are a direct effect of the surgical procedure rather than random variation. This evidence underscores the high clinical efficacy of cataract surgery in this patient population, supporting its continued use as a primary intervention to restore vision and improve quality of life (Table 2).

A one-way analysis of variance (ANOVA) was performed to evaluate whether postoperative visual acuity in the (OD), measured by LogMAR, differed significantly across predefined age groups. In this analysis, the dependent variable was the postoperative LogMAR visual acuity, and the independent variable was the categorical age group.

The ANOVA results showed a statistically significant effect of age on postoperative visual acuity, with an F-value of 1.386 (df_between = 51, df_within = 506) and a *p*-value of 0.045. Although the F-statistic indicates a modest ratio of between-group to within-group variance, the *p*-value below the conventional alpha level of 0.05 suggests that at least one age group’s mean postoperative visual acuity differs significantly from the others.

The sum of squares between groups was 5.999 and within groups was 42.935, reflecting the distribution of variance attributable to age group differences versus individual variability. The mean square values were 0.118 (between groups) and 0.085 (within groups), further supporting the observed variance pattern.

Since ANOVA indicates the presence of differences but does not specify which groups differ, post hoc pairwise comparisons (e.g., Bonferroni or Tukey HSD tests) are necessary to identify specific age groups with statistically different visual outcomes. This step is critical to better understand the influence of age on surgical success and to tailor clinical strategies accordingly (Table 3).

A one-way analysis of variance (ANOVA) was conducted to evaluate whether postoperative visual acuity in the left eye (OI), measured by LogMAR, differed significantly across patient age groups. The dependent variable was postoperative LogMAR visual acuity, and the independent variable was categorical age group. The results indicated no statistically significant differences in postoperative visual acuity among the age groups, with an F-value of 1.102 (df_between = 51, df_within = 506) and a *p*-value of 0.298. The sum of squares between groups was 5.425, while the sum within groups was 48.838, yielding mean squares of 0.106 and 0.097, respectively.

The *p*-value, substantially higher than the conventional significance threshold of 0.05, suggests that any observed variation in postoperative visual acuity across age groups is likely attributable to random chance rather than a true effect of age. Consequently, the analysis does not support the presence of meaningful differences in surgical outcomes by age in the left eye (Table 4).

**Figure 2:**
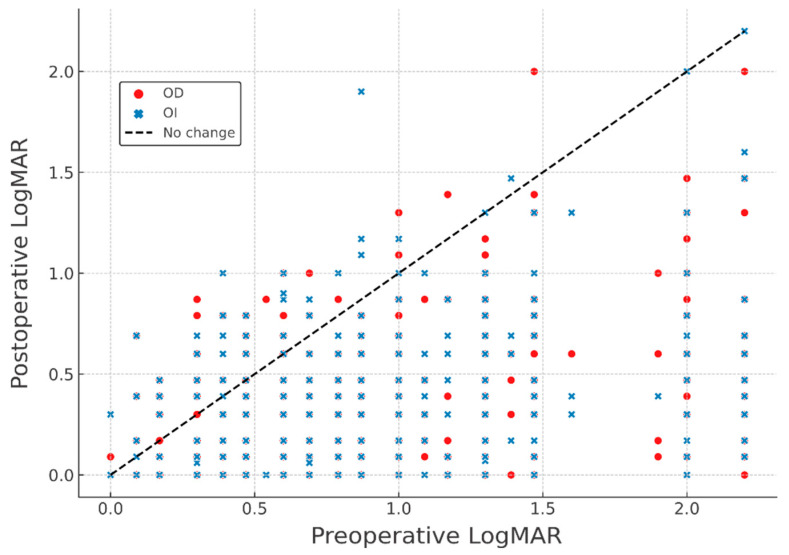
Scatter plot of pre- vs. postoperative LogMAR.

The scatter plot illustrates the visual impact of cataract surgery performed using the SICS technique by comparing visual acuity before and after the procedure in both eyes (OD and OI). Most data points lie below the identity line (y = x), indicating a generalized postoperative improvement. This trend is consistent in both right and left eyes, reflecting a symmetrical surgical response. However, some points appear above the line, suggesting that certain patients experienced a decline in visual acuity following the intervention. The predominance of points below the diagonal, along with the symmetrical distribution between OD and OI, supports the overall effectiveness of the procedure, although the cases of deterioration highlight the importance of comprehensive preoperative assessment and diligent postoperative follow-up (Figure 2).

In the logistic regression analysis of visual acuity in the left eye, the only sociodemographic variable that demonstrated a statistically significant association with visual outcomes was sex. Specifically, male sex was identified as a protective factor against poor visual acuity (≤1.0), with an (OR) of 0.572 (*p* = 0.036), indicating that males were less likely to experience poor visual acuity compared to females. The 95% confidence interval (CI) for this result was 0.339–0.964, confirming the significance of the finding, as the confidence interval does not include 1, indicating a lower likelihood of poor outcomes for males.

Among the age groups, the 40–49 group showed a trend towards an increased risk of poor visual outcomes, with an OR of 3.82. However, this result did not reach statistical significance (*p* = 0.066), meaning the observed effect could have been due to chance, and further investigation with a larger sample size could provide more clarity.

The other variables analyzed—age, area of residence, presence of diabetes mellitus, and arterial hypertension—did not show statistically significant associations with either the “regular” (>0.3) or “poor” (≤1.0) visual acuity categories. For instance, while diabetes and hypertension were prevalent, their presence did not significantly influence the visual outcomes in either category, suggesting that these conditions, at least in this sample, did not substantially impact the success of cataract surgery in terms of visual recovery.

Overall, these results indicate that male sex is associated with a lower risk of poor visual outcomes following cataract surgery, whereas the remaining sociodemographic factors (age, comorbidities, etc.) did not show a statistically significant impact on visual acuity. This finding suggests that, while other factors may influence cataract surgery results, sex (male) may be an important factor to consider when assessing visual recovery after surgery (Table 5).

In the logistic regression analysis for visual acuity in the right eye, a statistically significant association was found between age and regular visual acuity (>0.3), particularly in the 60–69 age group. This group had an (OR) of 2.663 (*p* = 0.002, 95% CI: 1.422–4.973), indicating that patients in this age range were significantly more likely to achieve favorable postoperative visual outcomes compared to the reference group (≥80 years). The statistical significance of this finding suggests that individuals in the 60–69 age group have a higher likelihood of experiencing better visual recovery after cataract surgery. Additionally, for poor visual acuity (≤1.0), although no other age group showed statistically significant results, it is important to note that the 30–39 age group had an implausibly small OR (1.12E−08), which resulted in a narrow confidence interval (0.000–0.000).

Furthermore, patients without diabetes were more likely to achieve regular visual acuity, with an OR of 1.624 (*p* = 0.018, 95% CI: 1.085–2.432), indicating that the absence of diabetes could play a protective role in achieving better visual outcomes. However, when examining the 40–49 and 50–59 age groups, the odds ratios were closer to 1, and the *p*-values were greater than 0.05, suggesting no significant association between these age groups and visual acuity outcomes.

Finally, no significant differences were observed for sex, area of residence, or hypertension in relation to postoperative visual outcomes. These findings reinforce the conclusion that age and the presence of diabetes mellitus are more influential factors in determining visual recovery following cataract surgery in the right eye. The significant result for the 60–69 age group particularly highlights the importance of considering age as a key factor in predicting postoperative outcomes (Table 6).

## 4. Discussion

The results indicate that cataract surgery significantly improves visual acuity, with an average gain of approximately half a LogMAR unit in both eyes. The age group with the highest surgical attendance was 70–79 years, followed by 60–69 years. Curiously, patients aged 60–69 experienced significantly better postoperative visual acuity in the (OD) than other age groups. Male sex emerged as a protective factor against poor visual acuity in the left eye. However, no significant associations were found for area of residence, hypertension, or diabetes mellitus. Although these comorbidities were highly prevalent, they did not significantly impact the postoperative visual recovery in either eye. Our findings support the efficacy of SICS and underscore the importance of age and sex as variables influencing the surgical outcomes of patients.

The results of this study are consistent with previously published evidence demonstrating the effectiveness and scalability of SICS in low- and middle-income countries. Previous randomized controlled trials and population-based studies have established SICS as a reliable, cost-effective technique for vision restoration, particularly suited for rural and resource-constrained settings [19,26]. For instance, a prospective study in Nepal comparing SICS with phacoemulsification found no significant differences in visual outcomes [27]. The SICS may be an affordable and logistically feasible option for large-scale surgical campaigns. Similarly, India’s Aravind Eye Care System has successfully implemented SICS across thousands of patients, reporting excellent postoperative visual recovery and low complication rates [28]. These global experiences underscore the value of adopting SICS as a national public health strategy in countries like Ecuador.

Our results reinforce the significant clinical benefits of SICS in improving visual acuity among older adult patients. SICS has previous global evidence supporting its efficacy as a cost-effective alternative to phacoemulsification [6,19], with comparable improvements in postoperative vision using SICS [15]. Moreover, these results align with the World Health Organization’s emphasis on scalable surgical interventions to reduce avoidable blindness in low- and middle-income countries [2,20]. The sustained visual improvements observed reinforce the role of SICS as a vital component of public health strategies in low–middle countries [19].

Previous studies have documented disparities in cataract surgery results and outcomes linked to sociocultural, biological, and healthcare access factors [29,30]. In our findings, male sex is linked to a lower likelihood of poor visual outcomes in the left eye, suggesting that sex-specific factors may influence recovery after surgery. Age is a determinant factor affecting postoperative recovery following SICS. Studies reports that middle-aged patients tend to achieve superior visual recovery due to reduced systemic decline, enhanced physiological resilience, and fewer comorbidities [31,32] Postoperative visual acuity in the (OD) differed significantly by age, with the 60–69 group exhibiting notably better outcomes than other age cohorts in our results. Finally, this study observed no significant relationship between diabetes mellitus or arterial hypertension and postoperative visual acuity. These findings contrast with previous research that identified metabolic disease or hypertension as comorbidity predictors of poorer surgical outcomes [33].

In comparison to other studies, the results of this study are in line with those reported in Nepal, India, and Latin America, but several methodological differences should be considered when interpreting the findings. One key difference lies in the timing of postoperative visual assessments. In the Nepalese study, visual outcomes were assessed over a longer follow-up period, which could explain the variation in visual recovery over time [27]. Additionally, while SICS was employed in both Nepal and India, minor differences in surgical techniques, such as the incision size and type of IOL used, could account for discrepancies in postoperative results [28]. Moreover, the patient populations in these countries differ from those in Ecuador, with Nepal and India often seeing a higher prevalence of comorbidities like diabetes and hypertension, which could affect the surgical outcomes. In contrast, the Ecuadorian population, while experiencing high rates of these comorbidities, did not show a significant impact on postoperative visual acuity, which may be explained by differences in comorbidity management and overall healthcare access [33].

Furthermore, studies from Latin America have documented variations in surgical outcomes due to differences in healthcare access, socioeconomic status, and the training of surgical teams [34]. These disparities may contribute to inconsistent results across different regions. For example, while SICS has been widely adopted in some Latin American countries, challenges such as the lack of postoperative care and delays in follow-up may affect long-term visual outcomes [31]. The study referenced from humanitarian campaigns involving SICS highlights another important point: the adaptability of the technique in settings with limited resources. The study in [35] emphasizes the use of SICS in humanitarian efforts, where the technique’s cost-effectiveness and ease of use are crucial in providing cataract surgery to underserved populations. This reflects the flexibility of SICS in both clinical and humanitarian contexts, further supporting its use in Ecuador.

This study has limitations inherent to retrospective multicenter analyses. First, reliance on preexisting clinical records limited the access to detailed patient histories, including information on ocular comorbidities, the type of IOL used, and the method of IOL power calculation. Second, the sample size, while adequate, may not fully capture the variability in surgical outcomes across different patient groups. Additionally, the absence of long-term follow-up data restricts our ability to evaluate the sustained effectiveness of SICS over time. Furthermore, data on cataract density, a critical factor influencing surgical difficulty and postoperative outcomes, was not available. The lack of patient-reported quality of life and functional vision post-surgery also limits a comprehensive assessment of the benefits of SICS beyond visual acuity alone. Nevertheless, this study represents the first multicenter assessment of cataract surgery outcomes in coastal Ecuador. The broad geographic coverage across six clinics provides a comprehensive view of surgical efficacy. Our results underscore SICS as a cost-effective, safe, and practical intervention for cataract blindness in low- and middle-income countries. Future prospective studies could address the identified gaps—including obtaining more detailed data on IOL types, cataract density, and long-term outcomes—and refine patient selection criteria. Ultimately, this work supports the expansion of SICS to reduce avoidable blindness in Ecuador.

## 5. Clinical Implications

This study highlights the effectiveness of SICS in improving visual acuity, especially in older adults, who benefit significantly from the intervention. The most relevant clinical implications include the recommendation to implement SICS in local and rural clinics, which could significantly increase access to cataract treatment and reduce the prevalence of avoidable blindness in Ecuador. Given that the Ecuadorian population experiences high rates of comorbidities such as diabetes and hypertension, but these did not significantly impact postoperative visual outcomes, it is suggested that public health programs focus efforts on optimizing preoperative and postoperative care, thereby minimizing complications. As demonstrated in this study, SICS could be an effective and cost-effective option in public health programs to reduce avoidable blindness, particularly in hard-to-reach areas.

Additionally, this study underscores the importance of strengthening the postoperative follow-up programs to ensure the long-term effectiveness of surgery, considering continuous improvement in patients’ quality of life. Public policies should integrate SICS into eye health campaigns, especially in rural contexts, so that this technique becomes accessible to the most underserved populations. Globally, this study is consistent with results obtained in countries such as Nepal and India, reinforcing the adaptability of SICS in different settings, including those with limited resources.

## 6. Suggestions

Future research should address the following key questions to further enhance our understanding and improve the outcomes of SICS:

Long-Term Visual and Functional Outcomes: What are the long-term visual and functional outcomes of SICS, especially in patients with comorbidities such as diabetes and hypertension? How does SICS compare with other surgical techniques over time, particularly in terms of complications like posterior capsule opacification and refractive errors?

Impact of IOL Types: How do different types of intraocular lenses (IOLs) (e.g., monofocal, multifocal, and toric) impact postoperative visual acuity in SICS patients? What is the effect of various IOL power calculation methods on postoperative outcomes and complications?

Cost-Effectiveness and Scalability: How does the cost-effectiveness of SICS compare to other cataract surgery techniques like phacoemulsification, particularly in low-resource settings? What are the barriers to accessing SICS, and how can these be overcome to improve its scalability and accessibility in underserved populations?

Patient-Centered Outcomes: How can patient-reported outcomes, such as quality of life and satisfaction with surgery, be incorporated into evaluating the success of SICS? What is the impact of SICS on patients’ ability to resume normal activities and overall life satisfaction?

Training and Skill Development: How can training programs for ophthalmologists in resource-limited settings be optimized to ensure high-quality SICS procedures? What training models, including simulation-based training, are most effective in improving surgical proficiency and outcomes?

## 7. Conclusions

This study is the first multicenter analysis in Ecuador to evaluate the efficacy of SICS in coastal Ecuador. In summary, our results demonstrated significant improvements in visual acuity following SICS, with an average gain of approximately half a LogMAR unit in both eyes. The highest surgical attendance was among patients aged 70–79. On the other hand, older adults aged 60–69 experienced significantly better visual outcomes in the right eye. Male sex was identified as a protective factor against poor postoperative vision in the left eye. Although diabetes mellitus and hypertension were prevalent in the population, these comorbidities did not significantly affect surgical outcomes. These findings confirm that SICS is a safe, effective, and affordable surgical option in resource-limited settings and highlight the importance of age and sex in influencing postoperative recovery. This study supports the broader implementation of SICS in low- and middle-income countries as a strategy to reduce avoidable blindness and enhance quality of life.

## Figures and Tables

**Table 1:** Sociodemographic data of participants included in the study.

Sociodemographic Background	*N* = 558 (%)	(χ^2^; gl; *p*-Value)	
		OD	OI
**Age**		20.03; 10; 0.029	17.81; 10; 0.058
30–39	4 (0.7%)		
40–49	12 (2.2%)		
50–59	46 (8.2%)		
60–69	161 (28.9%)		
70–79	260 (46.6%)		
80≥	75 (13.4%)		
**Sex**		0.375; 2; 0.829	4.27; 2; 0. 1118
Male	280 (50.2%)		
Female	278 (49.8%)		
**Zone**		0.37; 2; 0.0829	4.27; 2; 0.118
Rural	212 (38.0%)		
Urban	346 (62.0%)		
**HTN (hypertension)**		0.636; 2; 0.729	1.08; 2; 0.580
Yes	237 (42.5%)		
No	321 (57.5%)		
**Dbts (diabetes mellitus)**		4.49; 2; 0.106	0.85; 2; 0.653
Yes	370 (66.3%)	20.03; 10; 0.029	17.81; 10; 0.058
No	188 (33.7%)		

NOTE: χ^2^ = check chi-square; gl = degrees of freedom; *p* = statistical significance; % = percentage.

**Table 2:** Mean difference in LogMAR visual acuity before and after cataract surgery in the right (OD) and left (OI) eyes, using paired *t*-test.

		Mean	95% IC		t	gl	Sig. (Bilateral)
			Lower	Upper			
Pair 1	BEFORE CX (OD)–AFTER CX (OD)	0.5252	0.4837	0.5667	24.872	557	0
Pair 2	BEFORE CX (OI)–AFTER CX (OI)	0.4894	0.4481	0.5307	23.251	557	0

**Table 3:** One-way ANOVA comparing postoperative visual acuity in the right eye (OD) across different age groups.

	Sum of Squares	gl	Mean Square	F	Sig.
Between groups	5.999	51	0.4837	1.386	0.045
Within groups	42.935	506	0.4481		
Total	48.934	557			

**Table 4:** One-way ANOVA comparing postoperative visual acuity in the left eye (OI) among age groups.

	Sum of Squares	gl	Mean Square	F	Sig.
Between groups	5.425	51	0.106	1.102	0.298
Within groups	48.838	506	0.097		
Total	54.264	557			

**Table 5:** Multinomial logistic regression to identify predictors of regular and poor visual acuity in the left eye (AV OI).

Sociodemographic Background		Average (>0.3)			Bad (≤1.0)	
	Odds Ratio	95% CI	*p*-Value	Odds Ratio	95% CI	*p*-Value
		Lower Upper			Lower Upper	
**Age groups**						
(30–39)	593,216,596	199.45–542.3	0	3544	543.6–543.6	
(40–49)	0.265	0.030–2.349	0.233	3.82	0.915–15.951	0.066
(50–59)	1.027	0.451–2.341	0.949	2.19	0.730–6.574	0.162
(60–69)	1.592	0.870–2.912	0.131	2.084	0.851–5.104	0.108
(70–79)	1.23	0.704–2.149	0.466	1.388	0.590–3.268	0.453
80≥	Reference	Reference	Reference	Reference	Reference	Reference
**Sex**						
Female	Reference	Reference	Reference	Reference	Reference	Reference
Male	0.871	0.520–1.459	0.6	0.572	0.339–0.964	0.036
**Zone**						
Urban	Reference	Reference	Reference	Reference	Reference	Reference
Rural	0.744	0.508–1.089	0.128	1.179	0.706–1.967	0.53
**Dbts (diabetes mellitus)**						
Yes	Reference	Reference	Reference	Reference	Reference	Reference
No	1.243	0.832–1.857	0.287	1.226	0.702–2.140	0.474
**HTN (hypertension)**						
Yes	Reference	Reference	Reference	Reference	Reference	Reference
No	0.953	0.651–1.396	0.806	1.113	0.660–1.876	0.688

**Table 6:** Multinomial logistic regression to identify predictors of regular and poor visual acuity in the right eye (AV OD).

Sociodemographic Background		Average (>0.3)			Bad (≤1.0)	
	Odds Ratio	95% CI	*p*-Value	Odds Ratio	95% CI	*p*-Value
		Lower Upper			Lower Upper	
**Age groups**						
(30–39)	1.479	0.193–11.353	0.706	1.12E−08	0.000–0.000	-
(40–49)	0.802	0.180–3.569	0.772	1.35	0.289–6.313	0.703
(50–59)	1.604	0.688–3.741	0.274	1.774	0.659–4.778	0.257
(60–69)	2.663	1.4226–4.973	0.002	1.086	0.479–2.463	0.843
(70–79)	1.496	0.841–2.663	0.171	0.63	0.294–1.350	0.235
80≥	Reference	Reference	Reference	Reference	Reference	Reference
**Sex**						
Female	Reference	Reference	Reference	Reference	Reference	Reference
Male	0.956	0.664–1.377	0.81	1.1	0.646–1.874	0.725
**Zone**						
Urban	Reference	Reference	Reference	Reference	Reference	Reference
Rural	1.079	0.741–1.571	0.693	1.13	0.659–1.938	0.657
**Dbts (diabetes mellitus)**						
Yes	Reference	Reference	Reference	Reference	Reference	Reference
No	1.624	1.085–2.432	0.018	0.942	0.536–1.654	0.835
**HTN (hypertension)**						
Yes	Reference	Reference	Reference	Reference	Reference	Reference
No	1.006	0.690–1.466	0.977	1.173	0.678–2.031	0.568

## Data Availability

Data may be made available upon request. The original contributions presented in this study are included in the article. Further inquiries can be directed to the corresponding author(s).
